# Is the Invisibility of Dementia a Super-Power or a Curse? A Reflection on the SUNshiners’ Questionnaire into the Public Understanding of Dementia as an Invisible Disability: A User-Led Research Project

**DOI:** 10.3390/ijerph21040466

**Published:** 2024-04-10

**Authors:** Danielle Tingley, Rosalie Ashworth, Dalia Torres Sanchez, Grace Hayes Mac Mahon, Yvette Kusel, Brigitta Maria Rae, Tracey Shorthouse, Alan Bartley, Gabrielle Howell, Joanne Hurley

**Affiliations:** 1 SUNshiners Group, Kent and Medway Partnership Trust (NHS), Community of Mental Health for Older Adults, Folkestone Health Centre, Folkestone CT20 1JY, UK; 2 Neuroprogressive and Dementia Network, NHS Tayside, Ninewells Hospital, Dundee DD1 9SY, UK; rosalie.ashworth@nhs.scot

**Keywords:** invisibility, Dementia, co-production, user-led, mixed methods

## Abstract

The SUNshiners group includes people in the early stages of dementia with an interest in dementia activism and research. The group found that despite the growing awareness of invisible disabilities, there is very limited research into the pros and cons of the invisibility of dementia. Our paper explores the SUNshiners research which stemmed from varied individual experiences of disclosing diagnoses. The group designed and developed a short survey to explore what the public knew about dementia and what they thought about the invisibility of dementia. A mixture of open- and closed-ended questions were used to gain meaningful data. A total of 347 people completed the survey (315 online and 32 paper-based), which was then co-analysed. The findings suggest that the majority of the public felt that the invisibility of dementia was negative; that knowing someone had dementia when first meeting them would be beneficial; that people living with dementia should maintain the right to vote; and that people living with dementia do not automatically require a consistent, regular carer. Common themes from the open-ended answers included capacity, severity of dementia, and access to support. The findings support the disclosure of dementia diagnosis; however, more action is needed to tackle stigmatised views, particularly as the SUNshiners felt that people do not have enough dementia education to support a positive disclosure experience. They shared their experiences of the group and the project’s benefits, but also the losses they have faced. Our paper aims to be as accessible as possible.

## 1. Introduction

### 1.1. Co-Production

People have participated in research for over a hundred years, but such involvement has positioned participants as research subjects [1,2]. Social science research has explored methods that bring researcher and participant closer together, as with observation research [3,4] or action research [5,6]. These examples still maintain a relationship where the researcher holds the power and control over the research [7].

Some researchers have found that there is a “ceiling effect” to how much they can learn about people, without actively involving people with lived experience in the design and development of research [8].Further, there can be a reduction in trust and motivation in people with lived experience if they feel that they have been subject to research that has not been of benefit to them [9,10].

In recent years, there has been a growing interest in involving people with lived experience of dementia as partners in research [11,12]. Such involvement can be referred to as “co-production” or “patient and public involvement” [13]. The terms are often used interchangeably but capture a shift in power from being a research participant to becoming a co-researcher [14,15]. Examples of co-production in dementia include a decision aid in relation to end-of-life care [16] and the co-creation of “ageing playfully” resources [17]. Despite the growing drive for co-production in research, a literature review by Rivett [18] suggests that people with dementia are often still excluded from many areas of research and are rarely given the opportunity to be co-researchers. 

### 1.2. Dementia Enquirers

Co-research and user-led research are increasing [14]; however, there remains hesitation to adopt this approach from many despite recognition of the potential benefits [19]. More work is needed that highlights how user-led research can provide new, relevant knowledge that is of benefit to the scientific and wider community, as well as empowerment for those with lived experience [20]. There can be a stigma surrounding the effectiveness of this approach among some professionals [21], and in many ways, the hesitancy around user-led research has parallels with the adoption of qualitative research methods and social science more broadly [22,23]. To establish the scientific benefits of user-led research, we need more user-led research projects to be published within and outwith academia, with a commitment to explicit, transparent, and accessible research communication [24]. We also need to recognise that user-led values may not align with those of professionals, and researchers have an important role to play in bridging the different value-led systems [25,26].

The Dementia Enquirers project began in 2019; it recognises the value of “user-led” research by actively encouraging and supporting people with lived experience to be “in the driving seat” [27]. The project stemmed from growing frustration among people with lived experience that research was frequently conducted “about or to them” but not “with or led by them” [27]. The Dementia Enquirers project involves a core group of people with lived experience, known as Pioneers, supported by a small number of Advisors from the field of dementia [28]. The Advisors act as research “scaffolding” to support user-led research from the ground up, rather than top–down creation, as seen in other research areas [29].

The Pioneers from the Dementia Enquirers project were keen to show how the involvement of people with lived experience can generate new insights into topics that are of interest to people living with dementia [28]. Therefore, the Pioneers invited groups from the DEEP [30] network to submit research proposals. The applications included reference to a co-created accessible research pack [31]) and access to mentoring from the Advisors. Research proposals submitted to the Dementia Enquirers were reviewed and discussed in detail by the Pioneers before they chose a number of projects to fund. One such project was the SUNshiners [32].

### 1.3. SUNshiners

The SUNshiners are a DEEP group made up of 11 people living with early stages of dementia [30]. The group meet monthly and are involved in various activities, including working alongside the NHS health board to assist in training and service development; raising awareness of dementia; and engaging in research and research-related activities [33]. The group’s research topic arose from discussions about their everyday experiences, in particular the experience of people being surprised or disbelieving of their diagnosis. The positive and negative experiences of disclosure led to the question of whether the invisibility of dementia is helpful or not. Some of our members found the invisibility of their condition a relief, whilst others felt it led to disbelief or misunderstanding and thus found it a “curse”; hence, the title for our paper was created. The people living with dementia involved in this work were not subject to memory assessments or asked to provide in-depth details about themselves for the purpose of this project. Therefore, taking this approach helped group members to feel they were equals and leaders of the research. The eligibility to take part is assumed based on their group involvement in much the same way as academics are not asked to provide additional credentials within their publications. Taking measures to promote feelings of equal partnership can be seen in similar examples of co-research [34,35,36,37].

The research literature suggests that the invisibility of health conditions can delay access to appropriate diagnoses and support. For example, Olkin et al. [38] found that women with invisible disabilities were discounted based on the outward impression of health. However, this is not to say that disclosing a diagnosis or “making it visible” is the preferred approach [39]. Within research into disability and higher education, students with hidden disabilities faced frequent barriers but chose to hide their disability and not access support due to the stigma of the diagnostic label [40].

In relation to dementia, Truscott [41] draws attention to the difficulties a person with dementia faces when others think that they look well and therefore assume no illness. More recent research by Chaouni and De Donder [42] found that the invisibility of dementia can make it harder for people to recognise symptoms or signs of dementia, which then delays the ability to find the right care and support. Additionally, people living with dementia have not always been told their diagnosis, which adds to the invisibility [42]. Similar reflections have been seen from co-authors with lived experience in research by Ashworth et al. [43], with people wishing to have more choice in how and when to disclose a dementia diagnosis. Raising public awareness may empower more people living with dementia to disclose their diagnosis and give space for positive reactions [44].

Despite the potential benefits of visibility, in particular symptom recognition and access to services, there remains a very prevalent stigma surrounding dementia that can make people fear visibility or disclosure [39]. In support of this, Milne [45] noted that the word “dementia” is associated with many negative beliefs and assumptions (or stigma) that lead people to feel a need to hide their diagnosis. When the public have less accurate knowledge about dementia, it can be harder to challenge misheld beliefs, which keeps the cycle of stigma going.

A final consideration for the SUNshiners, and others interested in the invisibility of health conditions, was the introduction of the sunflower lanyard scheme [46]. In 2016, lanyards with sunflowers became a way for airport staff to support passengers with hidden disabilities. Over the course of the last 6 years, the scheme has expanded from airports into an international symbol of hidden disability, with over two million lanyards distributed. Despite the apparent success of the sunflower lanyard scheme, research into invisible disabilities such as autism has found mixed views on the scheme [47]. The availability of symbols to increase the visibility of invisible conditions supports the need for more information about whether this would be helpful for people living with dementia. Recent reflections from people living with dementia and carers suggest that there are clear individual differences in whether people use the lanyard, when, and for what purpose, highlighting the complexity around decisions to “display” disability [43].

The literature suggests that there are both positive and negative factors that shape how the visibility or invisibility of dementia impacts people with lived experience, for example, a risk of stigmatization [48] contrasting with a lack of recognition of symptoms [42]. Therefore, the SUNshiners group chose to conduct research which looked at whether the general public felt that the invisibility of dementia was positive or negative.

One of the barriers for people with dementia to lead on research is that they can be unaware of what research has already been conducted. Often, to access research articles, you either need to pay or be given access by an institution such as a university; however, there are access limitations for members of the public due to licensing agreements [49]. Given that people with dementia are often medically retired, gaining access to these articles is difficult, thus creating a wider gap between academia and the public and fuelling the frustrations of people with dementia who feel as though research is conducted “about them” not “with or led by them” [27,28]. To overcome the access barriers, the research literature was searched by academics supporting the SUNshiners work, who found that there was very little work available around the invisibility of dementia, as discussed in the previous sections. Overall, despite the examples above of work reflecting on invisibility and dementia, there was no research available that explored this topic on a larger scale and from the perspective of the public.

As part of the co-research, the SUNshiners shared some of their experiences in relation to the visibility, stigma, and public awareness of dementia. One SUNshiner was told by a member of the public that they “really needed to have a carer with them at all times”, when in fact they were able to live well independently. Their experience suggests that the invisibility of dementia could be positive, as people would not make false assumptions. In contrast, another SUNshiner informed airport staff of their dementia diagnosis and was offered excellent support when they arrived at the airport, supporting the positive side of making dementia visible. Contrasting experiences were also seen by two SUNshiners in hospital; one was given a separate, quieter room and allowed to have a family member present to support them, whereas the other was locked in due to a presumed fear that they would wander off. The following paper will demonstrate how a group of people with lived experience designed and developed a questionnaire to explore the invisibility of dementia and public misconceptions about dementia.

### 1.4. Aims

Our paper has two key objectives. Firstly, we aim to explore and reflect on the experiences of the SUNshiners when conducting user-led research. Secondly, we aim to contextualise the groups’ experiences by presenting the findings of user-led research where the SUNshiners explored some key beliefs around dementia among the public, with a particular focus on whether the invisibility of dementia is positive or negative.

### 1.5. Disclaimer

An initial report of the user-led research findings was provided to and published by the project funders, Innovations in Dementia [32]. We deliberately chose to include key information from this report in the following paper, as we feel that it provides important context to the wider discussion of co-produced research. We carried this out in accordance with publishing ethics. We recognise the risk of self-plagiarism [50,51] and therefore took steps to mitigate this. With the growing expectation to include people with lived experience in research, we need to continue to push for the findings to be available within an academic context, as well as within a public-facing report, in much the same way as academic research needs to be presented within a lay format.

## 2. Method (User-Led Questionnaire)

### 2.1. Participants

The questionnaire was open to people over 18 years of age who were living in the UK. A total of 347 people completed the questionnaire (315 online and 32 on paper). The majority of participants were female (78%), aged between 35 and 65 years old (61%), White British (88%), and had at some point had contact with a person living with dementia (62%).

### 2.2. Design

The research was conducted with a convergent parallel mixed-method design, whereby quantitative and qualitative data were collected in the form of closed and open-ended questions. The results were analysed separately before bringing them back together to answer the research questions that the questionnaire set out to answer. The design was chosen by the SUNshiners as the most suitable approach to answer their research questions in a timely fashion and with the aim of reaching as many people as possible. Their choice is in keeping with research to obtain opinions from the general public [52,53]. The choice of design, the questionnaire development, and analysis were supported by trained researchers using the scaffolding model set out by the Dementia Enquirers co-research guidelines.

### 2.3. Questionnaire Development

A standardised questionnaire on the invisibility of dementia does not currently exist; therefore, the SUNshiners created a bespoke questionnaire based on their lived experience and accessible language. The questionnaire was made available in two formats, online and on paper (please see Appendix A). The online questionnaire was hosted on SurveyMonkey and shared across the social media networks of NHS colleagues and DEEP members. Printed copies (*n* = 100) with stamped addressed return envelopes were placed in local shops, cafes, and social group locations so that people without computer access still had an opportunity to participate. The questionnaire had a total of 12 questions. The first 6 questions collected demographic information about participants, followed by 6 questions about dementia. Similar research has used online and print formats to improve access to dementia-related questionnaires [54].

**Figure 1:**
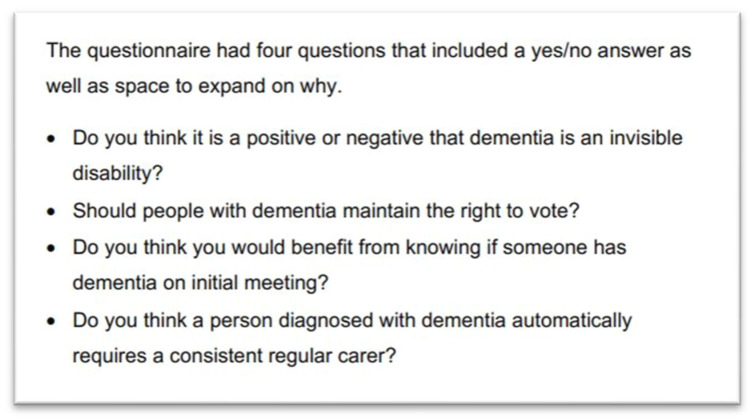
Four questions developed by the SUNshiners group as presented in the SUNshiners report.

The demographic questions also included asking participants what age they think someone can get dementia and to rate their knowledge of dementia from 1 to 10, with higher scores indicating higher self-rated knowledge. The questionnaire included both closed and open-ended questions, as seen in Figure 1.

The launch of the questionnaire was delayed to November 2021 in response to the pressure people were under with COVID-19.

### 2.4. Data Analysis

The SUNshiners approach to data analysis utilizes the scaffolding of support put in place through Dementia Enquirers to make sure expertise was available where needed. The volume of data that was collected was a challenge for the group. Whilst the SUNshiners were extremely motivated to achieve a high response rate from the survey, they were also keen to conduct this project themselves. However, most of the SUNshiners had little experience with data analysis, and with cognitive decline, it was much more difficult for them to focus on multiple answers whilst organising into multiple categories. Whilst they had the opportunity to trial the approach of managing both data and themes, feedback from them suggested that they were exhausted after sessions, which called for a modified approach to the analysis.

Due to there being no existing research available for conducting data analysis with people with dementia, the process was a collaborative process of negotiating and trialling methods. A descriptive analysis of the quantitative data was conducted, including demographic characteristics and self-rated knowledge of dementia.

The SUNshiners conducted a qualitative analysis of the open-ended data with support from researchers. A thematic analysis approach modified for the purpose of co-production was conducted, as described below.

The SUNshiners felt that the most effective method for them was to predict how the public may have answered. From this, pre-existing themes were created and Dr Ashworth supported this process by developing inclusion and exclusion criteria. This enabled the SUNshiners to pinpoint key terms for different themes, which made the process easier for them to manage. They agreed to categorise the first 30 answers for each question, and the Dr Ashworth was tasked with completing the others based on their analysis. This was later reviewed by the SUNshiners and agreed upon. By having fewer categories, it enabled the SUNshiners to effectively work with the data and see the development of new themes, which were passed back to the Dr Ashworth to further develop. A full report of the analysis criteria including examples is available in our lay report [32].

## 3. Results

### 3.1. Descriptive Statistics

A lay summary of the descriptive statistics is available in our SUNshiners report [32]. For the context of this report, we include the key quantitative and qualitative findings below.

The majority of people (83%) rated their knowledge of dementia as 50% or above. Almost half of the participants (45%) rated their knowledge of dementia as 8 or higher.

Non-parametric tests found a significant association between age of participant and self-rated knowledge (H(9) = 30.6, *p* = 0.01), with increased age associated with higher self-rated knowledge. A significant relationship was also found between whether someone currently has or previously had contact with a person with dementia and self-rated knowledge of the condition (H(9) = 50.5, *p* = 0.01), with contact associated with higher self-rated knowledge.

### 3.2. Age of Onset

The majority of people noted that dementia could develop at any age (60%), with the next most likely age being between 30 and 39. Of note, 15% of the answers included some awareness that age was a risk factor, with it being more common in older adults. There was not a significant difference between self-rated knowledge and age-of-onset rating (H(10) = 16.9, *p* = 0.08).

### 3.3. Research Questions

When asked whether it is positive or negative that dementia is an invisible disability, a significant majority of people (83%) felt that the invisibility of dementia is negative (χ^2^(1) = 178.3, *p* = 0.01). The remaining responses felt that the invisibility of dementia is positive (13%).

A significant majority of people (86%) also felt that people with dementia should maintain the right to vote (χ^2^(1) = 204.3, *p* = 0.01), with 11% of people answering that people with dementia should not maintain the right to vote.

A significant minority (22%) of people felt that they would not benefit from knowing if someone had dementia upon initially meeting them (χ^2^(1) = 320.0, *p* = 0.01), whereas 76% of people felt that it would be helpful to know if someone had dementia upon initially meeting them.

When asked if a person diagnosed with dementia automatically requires a consistent regular carer, the majority of people (78%) answered no, and a significant minority (20%) thought that a person diagnosed with dementia does automatically require a consistent regular carer (χ^2^(1) = 350.1, *p* = 0.01).

Across the four questions, there was an average completion rate of 97% for specifying answers.

Self-rated knowledge was considered in relation to the four open-ended questions, with no significant associations found. The outputs of the non-parametric testing are presented in Table 1.

### 3.4. Qualitative Answers

A summary of the questionnaire responses is provided below, with examples of the answers given in Table 2.

#### 3.4.1. “Do You Think It Is a Positive or Negative That Dementia Is Invisible?”

The majority of people thought that it was a negative that you cannot always see dementia as it can lead to a lack of understanding and a lack of awareness about symptoms and experiences. Not visibly having dementia can mean that the public are less aware of the condition and the need to learn more about it. However, focusing on people with dementia showing symptoms in order to help educate others puts a lot of the responsibility on the person with dementia. There is a sense that they should lose their privacy in order to help others understand what they are going through. Other answers put more of the responsibility on people without dementia. Across the positive and negative answers, the most prominent issue was that of the stigma surrounding dementia. From the answers, some saw the visibility of symptoms as a way to combat the stigma or a way of explaining symptoms so that they are not misunderstood. In contrast, others saw the stigma attached to dementia as a reason to keep symptoms invisible.

#### 3.4.2. “Should People with Dementia Maintain the Right to Vote?”

The majority of answers focused on whether a person had capacity. For people who said that people with dementia should not maintain the right to vote, there was a bigger emphasis on an assumed lack of capacity. Respondents raised concerns around whether a person with dementia would know what they were voting for and whether that could put them at risk of being manipulated. In contrast, for those who said that people with dementia should maintain the right to vote, there was a bigger emphasis on recognising that a diagnosis did not equal a lack of capacity and that people with dementia have a human right to vote and be an equal member of society. The answers across all respondents were very similar, but the difference came from whether people are assumed to have an ability or not.

#### 3.4.3. “Do You Think You Would Benefit from Knowing if Someone Has Dementia on Initial Meeting?”

The majority of answers centred on people wanting to know so that they could tailor their behaviour where needed to support someone better.

People were keen to know so that they could take responsibility for supporting a person with dementia and their needs, as opposed to expecting people with dementia to do anything differently. There was also an understanding that although it may be helpful to know, it was also a personal choice, with a right to privacy.

For people who thought it would not be helpful to know, privacy was the most common reason; the second most common reason was a tie between “should be kind and thoughtful to everyone regardless of diagnosis” and “may alter perceptions of behaviour”, which links back to concern about stigma.

#### 3.4.4. “Do You Think a Person Diagnosed with Dementia Automatically Requires a Consistent Regular Carer?”

Most people felt that the need for a consistent regular carer would depend on the severity of a person’s dementia. This was the case for people who answered yes and no. For people who thought that someone would require a carer, the answers were largely positive in that they thought it would be useful to have someone consistent and familiar involved in providing support, and that they were deserving of help.

The answers showed that people seemed aware of the individual differences and variation in capacity. The differences in whether people answered yes or no seemed to be linked to the assumed need for help; i.e., people answered yes if they thought people would need help vs. answering no if they thought people might need help.

## 4. Discussion

### 4.1. Questionnaire-Focused Discussion

The SUNshiners found that the majority of people completing the questionnaire had a good understanding of dementia based on self-rated knowledge. The majority of people recognised that dementia is something that could happen at any age, although it is more likely to be seen in older adults. It is positive to see a general increase in the public’s self-rated knowledge of dementia and the recognition that dementia can be early-onset [55].

Non-parametric tests did not find a significant association between self-rated knowledge and answers to the dementia-focused questions. In comparison, older participants and people who have known someone with dementia rated their dementia knowledge as higher than younger people and people who had not known someone with dementia.

Based on the questionnaire answers, we found that the majority of people felt that the invisibility of dementia was a negative thing. Most people felt that it would be beneficial to see dementia, which ties into how the majority of people felt that they would benefit from knowing that someone had dementia upon initially meeting them. Encouragingly, the qualitative answers evidenced that the reasons behind this were coming from a position of wanting to be able to help and provide appropriate support. However, for both answers, there is a question of how much stigma, assumed helplessness, and disempowerment could come from people labelling someone with dementia.

Both sets of answers people gave for whether someone should maintain the right to vote and required a consistent/regular carer centred on capacity and perceived risk. For those who felt they should not maintain the right to vote and/or do require a regular, consistent carer, they had assumed a lack of capacity. More work is needed to challenge the assumption of a lack of capacity [56]. Additionally, people living with dementia are likely to experience a loss of capacity at some point; therefore, it is important to understand their preferences around voting and carer presence so that one is not contemplating this for the first time when capacity is no longer there. Third-sector organisations are increasingly looking at topics such as capacity, human rights, and acceptable risk [57].

The findings suggest that people would prefer dementia to be a visible disability; i.e., it is a curse not a superpower. Such conclusions fit with that of the wider disability literature that people should feel empowered to disclose their diagnosis [58], as well as projects like the sunflower lanyard [59], where the aim is to have visible representations of the disability so that others can respond appropriately. Of course, disclosing an illness does not necessarily mean there will be understanding or acceptance [60]. The SUNshiners recognise that disclosure is dependent on the public’s understanding of dementia being accurate and compassionate. Research in other health areas has shown that even when public understanding of the biomedical nature of an illness improves, the stigma remains [61].

### 4.2. Limitations

The research used a non-standardised questionnaire, which can make it difficult to compare or generalise findings. However, the volume of data may also be a sign that the user-led design made the questionnaire easier for people to understand and complete. The questionnaire was also distributed to NHS colleagues, people living with dementia, the group’s social networks, etc., which may bias the sample towards people with a better understanding of dementia. The open-answer questions also required a binary answer before having space to expand. The findings also suggest that asking people just the binary question would not give a full enough picture, as for many, their qualitative answers highlighted a more context-dependent answer. For example, although the majority of people answered no to requiring a regular, consistent carer, the answers still recognised that people were deserving of such help if they wanted it, but it should be a choice not an assumed need.

### 4.3. SUNshiners Discussion

The SUNshiners reflected on their surprise regarding the number of people who completed the questionnaire, and how they had worried that “nobody was going to fill it in”, but in reality, they received a large number of responses, which was particularly difficult when planning analysis:

*“When all the answers came in, it was really difficult. Half way through a question we would forget what the question was, and it was never ending. We had 2 attempts at doing that when we realised it was too much for us.”*

It was not just the volume of responses that surprised the group but the fact that the majority of people thought it would be better if dementia was visible. In contrast, the group felt that they did not want dementia to be visible and shared various experiences of being either discriminated against and/or patronised when their diagnosis was disclosed. They also felt that the dementia diagnosis took away from all of their past identities, in keeping with the work around the “master status” of dementia [62]. Further, they felt that visible tools such as the sunflower lanyard rely on people knowing how to respond appropriately, but in general, they are more worried that they look vulnerable and are potentially at higher risk than if people did not know. The group discussed the need for more research into the education people have about dementia, so that if the diagnosis does need to be shared, they are treated appropriately. The most preferred option for if they did need to share their diagnosis was using a card that could fit in their pocket and be discreetly shared. Similar research around learning disabilities has encouraged a more co-designed lanyard process or a shifting of responsibility for staff to make themselves more visible as appropriately trained, rather than the individual having to disclose a diagnosis [63].

As well as reflecting on the questionnaire findings, it is also important to recognise the experience of people living with dementia taking on the researcher role to design, develop, and execute their questionnaire.

The success of the questionnaire and the contribution to the wider literature help to challenge assumptions that people living with dementia are not capable of getting involved in research. As discussed previously, there are limitations to the questionnaire when viewed from a position of standardized questionnaires and quantitative research validity. However, the questionnaire being created by people living with dementia may have made it more lay-appropriate and easier to fill in, as well as gaining a higher response rate. The data being collected by a group of people living with dementia may have helped people to feel less suspicious of answering the questionnaire, whereas academic researchers can often face this reaction [64]. The simplified questions also made it more likely that people living with dementia could answer the questions if they so wished [65]. We expected some respondents to be people living with dementia, as the SUNshiners have many connections within the dementia community who were likely to have seen the questionnaire. Researchers can often hold people living with dementia “at arms length”; in the kindest of cases, this is due to a fear of not wanting to overwhelm them. However, this often creates a further “us and them” divide between the two communities and leaves people living with dementia feeling frustrated that the research on their conditions does not always feel beneficial to them.

The experience of co-producing the data analysis also reinforces the benefits of scaffolding and incorporating expertise where needed, in line with more recent co-production research [66,67]. The limitations of the questionnaire discussed may have been alleviated if the questionnaire itself had been co-created, although the overall findings themselves may remain the same. The value of involving people with lived experience, as seen through user-led research, helps researchers interested in co-research to provide an evidence base around its validity and academic credibility, when accepting of the fact it cannot be viewed against the same benchmarks of quantitative research. As noted, this is in much the same way as the research community learning to evaluate qualitative research differently to quantitative research.

The SUNshiners reflected on how they found being involved in a research project on their topic of interest:

*“It made me feel like I was able to contribute to something positive by challenging members of the public to think about how it might feel to live with Dementia.”*

[32], p. 24

*“It was important to get involved in a research project for a few reasons, 1, to have a voice and 2, to prove to people that we can still do things. Just because we have a dementia doesn’t mean we are unable to get involved. Our brains still work to a degree just in a different way. I have enjoyed being part of the project and working as part of a team.”*

[32], p. 24

*“It has enlightened me positively to read the answers people gave. On the whole the answers were very caring in their responses. It also made me feel useful being part of the working group. I felt I had a purpose and enjoyed it very much.”*

[32], p. 24

It is important to recognise that although being involved in a research project can have many positive impacts, it also comes with an emotional toll. Dementia is a progressive condition which can have very individual differences in relation to the type of dementia, the speed of progression, and its symptomology: if you have met one person with dementia, you have only met one person with dementia.

Over time, greater difficulties with cognition and activities of daily living are expected. When working together as a group of people with lived experience, these changes are not experienced in isolation. One of the challenges the project faced was the pandemic. It was the decision of the SUNshiners to postpone the launch of their survey whilst the global pandemic was at the forefront of people’s lives. They did not feel people would have the capacity to think about dementia whilst the world was focused on COVID-19 and treatments for other disorders were paused. They therefore launched their survey in November 2021. Over the course of the SUNshiners project, and in part due to the extended time needed for the work over COVID-19, group members experienced changes in their health. Sadly, one group member passed away, and another member was no longer able to get involved.

*“The sad thing for me is that you make friends in this group and then seeing them go down or disappear completely as the illness grabs them, it is really sad.”*

In the end of project meeting, the group reflected on their loss and the complexity of grieving for those you have cared about, whilst also reflecting on your own future prognosis. It is important that groups such as this have the appropriate support in place to be able to manage these experiences alongside their research activities. More research into how to support people through their experiences of loss is needed.

## 5. Conclusions

The SUNshiners group designed, developed, and executed a questionnaire that over 300 people responded to. They found that the majority of people felt that the invisibility of dementia was negative and that it would be helpful to know if someone had dementia when meeting them, because then you can understand symptoms and provide support. The public recognised that people living with dementia should maintain the right to vote and a diagnosis does not mean that an automatic regular carer is needed; however, they acknowledged that this was largely depending on the individual and their symptoms.

The findings suggest that the public understanding of dementia is improving and that there is a wish to be able to provide appropriate support. However, although we know that the public would prefer dementia to be visible, the reflections from the SUNshiners group show that they do not feel confident that the stigma has reduced enough to feel comfortable. More research is needed into whether people living with dementia would prefer to be visible and to what extent (i.e., automatic sunflower lanyards or a more dementia-specific symbol?), as well as research into supporting co-production groups through the loss and change of group members as well as within themselves.

Finally, the SUNshiners project and its experience of co-production provide an important example of how user-led research can contribute to the scientific community.

## Figures and Tables

**Table 1:** Non-significant relationships between self-rated knowledge of dementia and the four research questions.

Association with Self-Rated Knowledge of Dementia	Non-Parametric Test Output
Do you think it is a positive or negative that dementia is an invisible?	H(9) = 5.4, *p* = 0.80
Should people with dementia maintain the right to vote?	H(9) = 4.8, *p* = 0.85
Do you think you would benefit from knowing if someone has dementia on initial meeting?	H(9) = 12.0, *p* = 0.21
Do you think a person diagnosed with dementia automatically requires a consistent regular carer?	H(9) = 8.9, *p* = 0.45

**Table 2:** Examples of open-ended responses to SUNshiners questionnaire.

Question	Type of Answer	Comments
**“Do You Think It Is a Positive or Negative that Dementia Is an Invisible?”**	Negative	“Any hidden illness causing extreme extra pressures such as lack of understanding of additional needs at events.” “As it can’t be seen people don’t make allowances for it and don’t understand what others are going through.” “It is very socially impairing but being ‘invisible’ means that others won’t be prepared or recognise it.”
	Positive	“People think that you are stupid when you can’t recall the simplest of things.” “Labels can sometimes cause more problems than solving them. We should all have an awareness about what happens to us in life and accept and understand these things as a normal part of life.”
	Neutral	“It [the invisibility] can be a positive or a negative. It is not easy for people to see the disability then they cannot make allowances. However if you are the person with dementia it may be a positive as you may not want people to know at certain times.” “I think it could be both to be honest. Positive because people can claim for extra help and support but it could also be seen as a negative label.”
**“Should People with Dementia Maintain the Right to Vote?”**	No	“May lead to influence being put on them by others.” “They may be pushed into voting for someone they don’t want.” “They cannot retain the necessary information to make an informed choice. Those with mild dementia may reflect on political mandates from many years ago which may not be the policies of current candidates. Those with severe dementia would need to be told to cross the box and which one so it basically gives 2 votes to the carer.”
	Yes	“Mental capacity is often maintained for a long time after a diagnosis of dementia.” “Equality under Human Rights Act as long as they have mental capacity.” “Because people living with dementia do not necessarily lack capacity or the capability to make informed decisions.”
	Neutral	“As long as they have capacity, understanding what is going on.” “Only if at the time of voting they have clear understanding of their vote.”
**“Do You Think You Would Benefit from Knowing if Someone Has Dementia on Initial Meeting?”**	Yes	“It would help me adjust communication style to ensure information is given in small chunks, repeated often and written down.” “It would explain certain behaviours and more patience and understand could be used.” “Mostly, not always. It gives people a cue that they may need to take a bit of extra consideration with that person.”
	No	“Once a person with dementia says they have a dementia they are treated very differently. Nobody should be treated differently due to their health status.” “I don’t need to know if someone had ulcerative colitis or diabetes so why would I?” “People are defined by more than their diagnosis.”
	Neutral	“yes and no. If people want to share fine, but people also have the right to keep it private.” “Because it would help them adapt their interaction with them and set better expectations for it. But it depends on the purpose and context of the meeting, I don’t think it should be a blanket yes.”
**“Do You Think a Person Diagnosed with Dementia Automatically Requires a Consistent Regular Carer?”**	No	“Dementia does not define need/everyone is different.” “Carers CAN limit capability and increase speed of decline.” “People living with dementia are not in need of care all the time, they may need extra support but are capable of living independently.”
	Yes	“People deserve access to help.” “Consistency is a big help. Only needs a carer at certain stages but familiar faces are very important to ease confusion!” “It helps to build trust in a ‘safe’ person for when the disease reaches the later stages and the patient is not always able to make their wishes and needs known.”
	Neutral	“I guess there are varying degrees.” “Depends on the individual need.”

## Data Availability

Data will not be provided due to NHS data privacy restrictions.

## Appendix A

**Information Sheet**

The purpose of this nationwide study is to investigate the public’s perception regarding the invisibility of dementia. The study contains a short survey which will take no longer than 5–10 min to complete. The data collected will not identify individuals and it will only be used for the purpose of this study. Your responses will be very much appreciated. There will be links at the end of the survey if you require any further information.

We do ask that you only complete the survey is you are over the age of 18 and are a UK resident—thank you.

**Consent Form**

By filling out this survey you are consenting to the following:

I confirm I have read the information sheet for the above study

I understand that the data collected will be anonymised

I understand that the data collected will be used for research purposes only

I agree to take part in the study

**Demographic questionnaire:**
**What gender are you?**

☐ Male

☐ Female

☐ Other

**How old are you?**

☐ 18–25

☐ 26–34

☐ 35–50

☐ 51–65

☐ 66–75

☐ 76+

**What is your Ethnicity?**

☐ Asian (Bangladeshi, Chinese, Indian, Pakistani, Other)

☐ Asian British

☐ Black (African, Caribbean, Other)

☐ Black British

☐ Mixed ethnicities

☐ White (American, European, Irish, Other)

☐ White British

**What county do you live in?**
**Do you have or have contact with a person living with dementia?**

☐ Yes

☐ No

**How would you rate your understanding of dementia? (add circle)**

1 → 2 → 3 → 4 → 5 → 6 → 7 → 8 → 9 → 10

Little understanding → Good understanding

**Main questionnaire**

How old do you have to be to have dementia?

**Do you think it is a positive or negative that dementia is an invisible disability?**

☐ Positive

☐ Negative

**Please explain why:**
**Should people with dementia maintain the right to vote?**

☐ Yes

☐ No

**Please explain why:**
**Do you think you would benefit from knowing if someone has dementia on initial meeting?**

☐ Yes

☐ No

**Please explain why:**
**Do you think a person diagnosed with dementia automatically requires a consistent regular carer?**

☐ Yes

☐ No

**Please explain why:**
